# Butyrylcholinesterase levels correlate with surgical site infection risk and severity after colorectal surgery: a prospective single-center study

**DOI:** 10.3389/fsurg.2024.1379410

**Published:** 2024-08-20

**Authors:** Georgios-Ioannis Verras, Francesk Mulita

**Affiliations:** ^1^ Department of General Surgery, University Hospital Southampton, NHS Trust, Southampton, United Kingdom; ^2^ Department of Surgery, General University Hospital of Patras, Patras, Greece

**Keywords:** butyrylcholinesterase, inflammation, prediction, surgical site infection, colorectal surgery

## Abstract

**Introduction:**

Surgical site infections (SSIs) after colorectal surgery remain a significant concern, which warrants effective predictive markers for prompt diagnosis and treatment. Butyrylcholinesterase (BChE), a non-specific cholinesterase enzyme, has been correlated with the risk of hepatic dysfunction progression and, more recently, infectious diseases and septic shock with ongoing research into the utility of BChE in multiple systemic inflammatory conditions. Whether these preliminary results can be translated into predicting infection after colorectal surgery remains in remains in question. This prospective study aimed to assess BChE's potential as a predictive marker for surgical site infections and anastomotic leaks after colorectal surgery.

**Materials and methods:**

This single-center prospective study (11/2019–05/2023) enrolled 402 patients who underwent colorectal surgery. BChE levels were measured at four postoperative time points. The primary endpoints focused on BChE's association with complications, particularly surgical site infections (SSIs). Further known predictors of SSI were utilized to construct multivariable models to assess for independent association with SSI development.

**Results:**

During the third and fifth day postsurgery, SSI patients had significantly lower mean BChE levels (3.90 KU/L vs. 4.54 KU/L *p*-value < 0.05, and 4.14 KU/L vs. 4.73 KU/L, *p*-value < 0.05; *t*-test, respectively). However, multivariate analysis revealed that when adjusted for other factors, low BChE levels on the first postoperative day were associated with 2.6 times higher odds of developing SSI (OR: 2.6, 95%CI: 1.3–3.9, *p-*value < 0.05). Similar results were found for low BChE levels on the third postoperative day as they were associated with a. 2.53 times higher odds for developing SSI (OR: 2.5, 95%CI: 1.27–3.87, *p*-value < 0.05) when adjusted for other factors.

**Conclusion:**

In conclusion, in this prospective observational study, low levels in the first and third postsurgery were associated with an increased risk for the development of SSIs but not sepsis.

## 1 Introduction

Colonic resections during colorectal surgery are generally associated with high rates of infectious complications, notably surgical site infections (SSIs) (1–11). SSIs constitute approximately one-quarter of all hospital-acquired infections, affecting up to 5% of all surgical patients, with one-fourth of those cases reported after colorectal surgery (1–11). SSI post-colorectal surgery is associated with poor prognosis, increased mortality rates, lengthier hospitalization, and up to threefold increase in hospital costs making it a major healthcare challenge (5, 7, 12–16).

To confront this, both the American College of Surgeons and Surgical Infection Society and the World Health Guidelines recommend prophylactic antibiotic therapy for SSI prevention in high-risk patients (1, 2, 12, 17, 18). Butyrylcholinesterase (BChE) is an alpha-glycoprotein present in most tissues, particularly in the liver. Lower BChE levels have been linked with increased mortality in liver transplant surgery. In addition, terminal ill cancer is accompanied by mild to moderate inflammation and various degrees of protein–energy malnutrition (PEM), resulting in reduced plasma BChE levels and increased mortality risk (19). Moreover, contemporary data from retrospective observational studies report that low BChE levels are independent predictors of severe systemic inflammation with this phenomenon occurring early in the inflammation cascade. This phenomenon raises the possibility of minimizing the time delays between the clinical assessment and treatment of the underlying inflammatory process factors such as SSI. However, to date, there is paucity of data concerning the translation of these data to colorectal cancer surgery patients.

This study aimed to evaluate BChE as a potential marker for the risk of developing SSI and septic complications in patients undergoing colorectal surgery.

## 2 Materials and methods

### 2.1 Study design

This prospective single-center study was conducted according to the STROBE statement and recruited consecutive patients undergoing colorectal surgery from November 2019 to May 2023 in an academic tertiary hospital in Greece. Patients were enrolled in the study after providing informed consent. The study protocol was approved by the hospital's Ethical and Scientific Review Board (Approval No. 42687/0519) and was registered in an open-access database available on the Internet (www.clinicaltrials.gov: NCT04748744).

### 2.2 Inclusion and exclusion criteria

In this study, data were evaluated from 403 consecutive patients who underwent colorectal surgery at the Surgical Clinic of the General University Hospital of Patras (GUHP). Patients were included in the study, provided they had completed the necessary informed consent documents after a counseling session with members of the research team. In these sessions, the purposes of the study, the research perspective, the interventions to which they were to be subjected, as well as the fact that their participation is voluntary and they retain the right to withdraw from it at any time, even after its end, until the publication of its results, were analyzed to the patients. Patients were included in this study if they met the following inclusion criteria: (1) age 18 years and older, (2) ability to undergo surgical intervention on an urgent or an elective basis, and (3) requiring colorectal surgery for any surgical pathology. We included both elective and urgent/emergency operations, as they will be further analyzed as separate subgroups in the analysis. Due to the effects of systematic inflammatory states and neurological degenerative disorders in BChE levels, we decided to exclude patients who exhibited signs of metastatic disease, either localized or with extended metastatic disease burden.

In total, 489 patients were screened for inclusion in the present study. Of them, 67 were deemed unfit for surgery, or their management plan was switched to conservative management before undergoing surgery, and 19 patients were not able to provide consent at the time of operation or in the early postoperative period.

### 2.3 Serum BChE measurement

During hospitalization, patient serum samples were obtained at four time points: (1) on the day of surgery, preoperatively, and a second sample immediately postoperatively, (2) first postoperative day, (3) on the third postoperative day, and (4) on the fifth postoperative day.

The reasoning behind this measurement protocol was to incorporate preoperative (baseline) measurements of BChE within our modeling process. For the measurement of BChE, materials and resources of the GUHP were used, which had been approved at the time of study submission. The quantitative *in vitro* determination of BchE in the serum was done using a colorimetric method. For the determination of BChE levels, a spectrophotometric method with a Randox RX Imola Autoanalyzer was used. The values were expressed in IU/L (international units per liter). The values of BChE levels, as well as all the patient data concerning their hospitalization, were recorded in a special postoperative monitoring software of the clinic, transferred to separate databases, and anonymized before the analysis phase. As part of the project's protocol, the biological patient samples were kept until the end of the analysis phase (September 2023), after which they were appropriately disposed of and destroyed without storing any tissue samples.

### 2.4 Study endpoints and outcome measures

Our primary endpoint was the development of surgical site infections (SSIs), as a subcategory of septic complications. SSIs were defined using the CDC criteria for diagnosis and classified in accordance with the CDC/NSQIP classifications of SSIs. SSIs were classified as superficial incisional SSIs, deep incisional SSIs, and organ/space SSIs (20, 21).

As a secondary endpoint of our study, we set the development of any postoperative septic complication, with a subset analysis on patients whose septic profile was secondary to an anastomotic leak.

For the definition of septic syndromes, the latest definition according to Sepsis-3 was used: sepsis should be defined as life-threatening organ dysfunction caused by a dysregulated host response to infection. For clinical operability, organ dysfunction can be represented by an increase in the Sequential Organ Failure Assessment (SOFA) score of 2 or more points, which is associated with in-hospital mortality greater than 10%. For this composite measure, all septic complications were considered, including but not limited to anastomotic leaks, surgical site infections (of all grades), hospital-associated pneumonia, UTIs, and more.

As a special patient subpopulation of interest, patients with septic syndrome secondary to anastomotic leaks were studied separately. The diagnosis of the leak was made using clinical indicators and was radiologically confirmed in all cases as per local workup protocol. These three outcomes were studied in relation to age, gender, preoperative diagnosis of the patient, the degree of urgency of the surgery, the presence of malignancy, the duration of the operation, and the number of pRBC units transfused.

As a final secondary outcome of the study, we set the development of any postoperative complication, as defined by the ESA-ESICM joint task force on perioperative outcome measures. The existence or not of a postoperative complication, and its categorization, was made after the evaluation of the patient by at least two doctors at the time of diagnosis, and any disagreements as to the definition and identification of it were resolved by a senior, third specialized colorectal surgeon.

All patients received a bundle of SSI prophylaxis based on the 2019 NICE guidelines for the prevention of SSIs. Patients in our institution received preoperative antibiotics within 30–60 min from the first incision. Appropriate warming, hair removal, and glycemic control were ensured throughout the procedure. Skin preparation included alcohol-based chlorhexidine solution for all patients. Elective colorectal patients received intravenous cefuroxime and metronidazole. Emergency cases routinely received beta-lactamase plus metronidazole; however, ciprofloxacin was also utilized in some cases. In our institution, mechanical bowel preparation is used routinely in elective cases. Oral antibiotics in elective colorectal cases are not part of the institution's protocol and therefore were not administered (20).

### 2.5 Statistical analysis

For the statistical analysis of the results of the work, the statistical data processing packages SPSS (IBM SPSS Statistics for Windows, Version 28.0. Armonk, NY, USA: IBM Corp), jamovi [The jamovi project (2021). jamovi (Version 1.6)], and the R programming language were used. The variables of interest were expressed as binomial (binomial variables) and concerned the development or not of SSI, the development or not of postoperative sepsis, and the presence of anastomotic leakage, as a cause of postoperative sepsis. The presence or absence of malignancy, gender, operative approach, urgency on which the patient was operated, smoking, and the ASA score greater than 2 were also expressed as binomial. Preoperative diagnosis, type of surgery, TNM staging, and the number of RBC units administered were expressed as categorical variables. The days of hospitalization, age, BChE levels in the blood, BMI, and surgical time in minutes were the continuous variables. At the same time, some continuous variables were deemed necessary to be transformed either logarithmically or to the root of the variable to meet the requirements of the regression models.

The initial approach to the data was made using univariate analysis techniques for pairs of variables to establish the existence of any associations between them. For the comparison of continuous variables, the means for each category and the standard deviations were checked for statistical significance using either *t*-tests (Student's) or Mann–Whitney *U*-test when the use of parametric tests was not feasible. The normality of data was evaluated by using visualization of the variable distribution (histograms) and with the utilization of the Shapiro–Wilk test. For the comparison of categorical variable values, Fischer's exact test and the Chi-squared test of statistical significance were used as appropriate. For all tests of statistical significance, *p* < 0.05 was considered the threshold of significance, while all tests were two-tailed.

As the final step in the statistical analysis of the study data, the construction of a predictive model for the outcomes of interest was defined. For the creation of the predictive models, we relied on the principles of logistic regression modeling. The stepwise selection regression technique was used for the selection of parameters and manual parameter selection to achieve optimal model fit.

To assess the weighed and independent utility of BChE in assessing SSI patients, multivariate logistic regression analysis using backward variable selection techniques was used. The optimal logistic regression model resulted from both manual extraction and insertion of certain variables, determined by the reported AIC. After constructing the original model, we utilized the omnibus likelihood ratio test to assess for each model whether the variance explained by the model in the observed data is statistically more significant than the unexplained variance. We selected the variables that proved to be independently associated with SSI, to construct the final predictive model. To assess the final model fit, McFadden's pseudo-*R*^2^ test was employed.

For the evaluation of the goodness of fit of the predictive models, on our data, the Akaike information criterion and the *R*^2^ indices according to McFadden's pseudo-*R*^2^ test were employed.

Finally, after construction of the optimal model, we evaluated its ability to predict the occurrence of SSIs by using bootstrapping sample drawing and plotting the corresponding ROC curve.

## 3 Results

**Figure 1:**
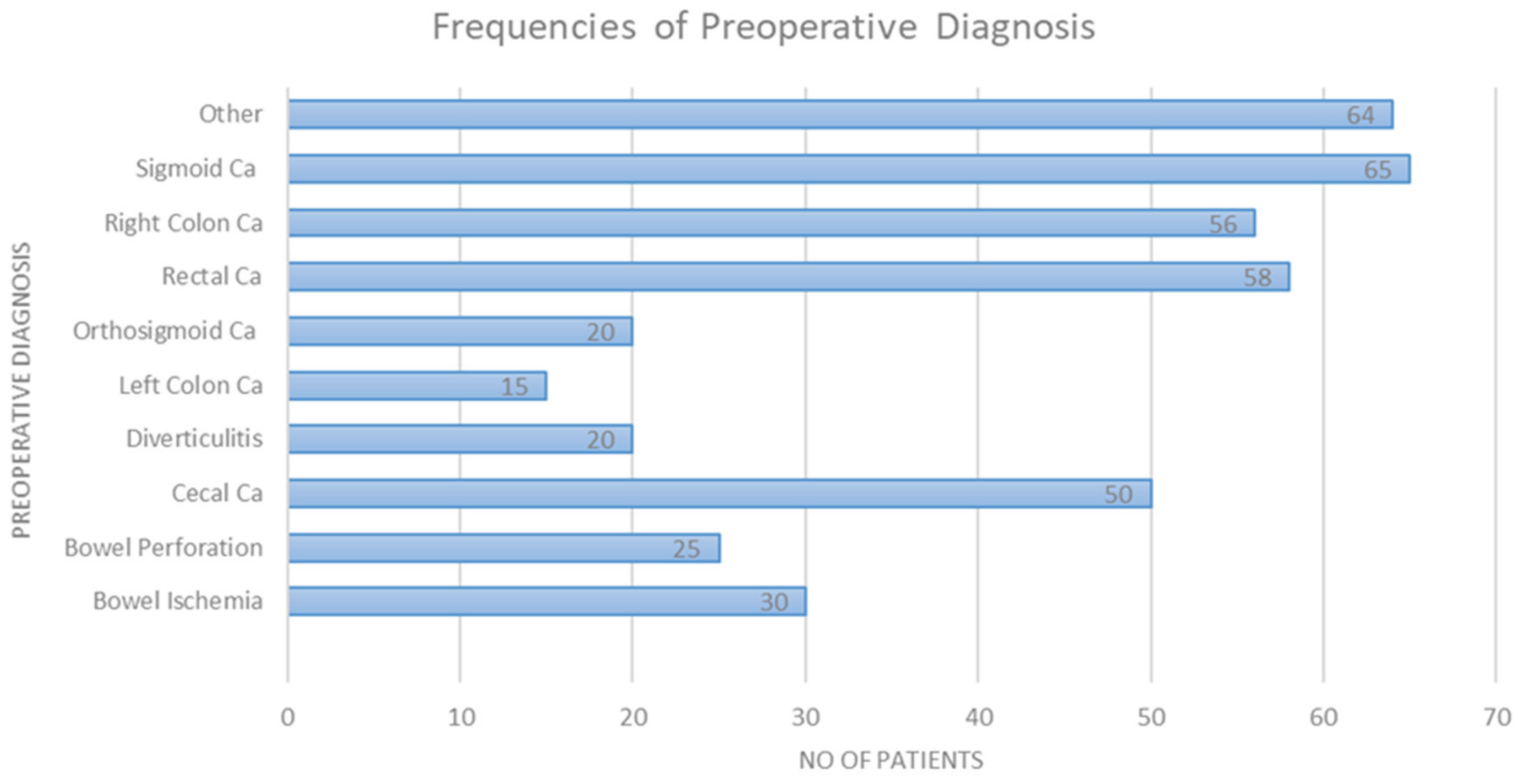
Preoperative patient diagnoses.

The study included a total of 403 patients of which 226 were males (56.2%). The presence of malignancy constituted the most significant category of preoperative diagnosis, with a prevalence of 71.6% among the patients. Sigmoid colon malignancy accounted for 16.2% of the preoperative diagnoses. The surgeries performed were (in descending order) right hemicolectomy (39.3%), anterior resection (17.9%), low anterior resection (11.2%), and Hartmann’s sigmoidectomy (10.2%). Most surgeries were performed with an open approach (87.8%). Of note, this was attributed to the percentage of patients urgently directed to surgery, constituting 30.6% of cases. Table 1 outlines all demographic patient characteristics. Figure 1 illustrates the preoperative diagnoses of enrolled patients, and Supplementary Table S1 outlines them in detail.

**Table 1:** Baseline patient characteristics.

Factors	No. of patients	Total percentage (% )	*p*-value
Presence of malignancy			0.405
Malignancy	288	71.6	
No malignancy	114	28.4	
Gender			0.064
Male	226	56.2	
Female	176	43.8	
Operative approach			<0.01
Open	353	87.8	
Laparoscopic	49	12.2	
Urgency of operation			0.041
Elective	279	69.4	
Urgent/emergency	123	30.6	
ASA score			<0.01
ASA score <2	287	71.4	
ASA score >2	115	28.6	
Smoking			0.12
Smoking	73	18.1	
Not smoking	330	81.9	
Diabetes mellitus			0.002
Diabetes	89	22.08	
No diabetes	314	87.92	
BMI	** **	** **	0.004
Less than 30	129	32.0	
More than 30	274	68.0	
pT			0.284
T1	19	4.71	
T2	193	47.8	
T3	162	40.1	
T4	29	7.19	
pN			0.175
N0	271	67.25	
N1–N3	132	32.75	
AJCC eighth stage			0.231
I	182	45.16	
II	72	17.86	
III	149	36.97	

### 3.1 Complications and surgical site infection rates

Among the remaining patients, 15.2% (61 patients) experienced surgical site infection (SSI) as the predominant postoperative complication. In addition, 6.5% (26 patients) developed any form of postoperative infection, while 4.2% (17 patients) developed septic complications, primarily due to postoperative leakage from a newly formed anastomosis. Most of the enrolled patients (69.7%) had a complication-free early postoperative period. The development of any complication affected a total of 122 patients, which corresponds to 30.3% of the participants. SSI as a complication affected 61 patients (15.2%).

### 3.2 Univariate analysis—surgical site infections

Operative approach, urgency of operation, ASA grading, preoperative BMI, and diabetes mellitus were all found to be significantly associated with the occurrence of SSI in the univariate analysis (Table 1). These are the parameters that multivariable analysis and model building will be based on; however, crucial parameters such as sex and age will also be included based on previous larger studies that have documented a strong relationship between them and SSI.

### 3.3 Serum butyrylcholinesterase levels at different time points between patients with SSI and uncomplicated patients

Looking at Table 2, we can observe that patients with surgical site infection (SSI) tend to differ from uncomplicated patients in terms of the mean BChE levels in their blood serum. On the day of surgery and the first postoperative day, patients with SSI had higher BChE levels on average (5.41 KU/L vs. 5.16 KU/L, *p* = 0.164 and 4.69 KU/L, vs. 4.61 KU/L, *p* = 0.658, respectively). This trend reverses on the third and fifth postoperative days, where patients who developed SSI had significantly lower serum BChE levels on average (3.90 KU/L vs. 4.54 KU/L, *p* < .001 and 4.14 KU/L vs. 4.73 KU/L, *p* < .001, respectively). Preoperative (baseline) serum BChe levels did not differ significantly between patients.

**Table 2:** Univariate analysis of serum BuChE levels in SSI.

BuChE value	Group	Mean	SD	*p*-value
BuChE (day of operation)	SSI	5.41	1.32	0.194
	Uncomplicated	5.16	1.28	
BuChE (first postoperative day)	SSI	4.69	1.27	0.658
	Uncomplicated	4.61	1.25	
BuChE (third postoperative day)	SSI	3.90	1.06	<0.001
	Uncomplicated	4.54	1.20	
BuChE (fifth postoperative day)	SSI	4.14	1.06	<0.001
	Uncomplicated	4.73	1.22	

### 3.4 Multivariate analysis

Multivariate analysis revealed that when adjusted for other factors, lower BChE levels on the first postoperative day were associated with 2.6 times higher odds of developing SSI (OR: 2.6, 95%CI: 1.3–3.9, *p-*value < 0.05). Similar results were found for low BChE levels on the third postoperative day as they were associated with 2.53 times higher odds for developing SSI (OR: 2.5, 95%CI: 1.27–3.87, *p*-value < 0.05) when adjusted for other factors. Lastly, when adjusting for other factors, BChE levels on the fifth day were not independent risk factors for SSI development (OR: 0.38, 95%CI: 0.02–1.23, *p*-value > 0.05). All the above results were obtained, using baseline BChE measurements on the operative day, as the reference level (Table 3).

**Table 3:** Multivariate analysis and predictive model metrics for the development of SSI.

Predictor	Estimate	SE	*Z*	*p*	Odds ratio
Intercept	1.8451	1.6092	1.1466	0.252	6.329
BuChE (first postoperative day)	−11.3132	1.8003	−6.2840	**<.001**	**2**.**6**
BuChE (third postoperative day)	11.4673	2.1995	5.2135	**<.001**	**2**.**53**
BuChE (fifth postoperative day)	1.0813	0.9687	1.1162	0.264	0.38
Gender					
M—F	0.1302	0.6167	0.2111	0.833	1.139
Age categorical					
Under 65–65 or older	−0.0377	0.6323	−0.0596	0.952	0.963
ASA score					
<2 ->2 (reference)	−1.9801	0.6512	−3.0407	**0**.**002**	**0**.**138**
Malignancy/no malignancy					
No malignancy—malignancy (reference)	−1.6631	0.6568	−2.5319	**0**.**011**	**0**.**190**
Hospital stay (days)	−0.0296	0.0466	−0.6366	0.524	0.971
Model fit measures					
**Model**	**Deviance**		**AIC**		**R²_McF_**
1	79.1		97.1		0.735
Predictive measures					
**Accuracy**	**Specificity**		**Sensitivity**		**AUC**
0.952	0.852		0.978		0.981

Entries in bold indicate statistical significance.

An ASA score of less than 2 was an independent negative predictor of SSI occurrence (Table 3, with an OR of 0.138). Malignancy status was also an independent predictor of SSI, with an OR of 0.190 (no malignancy vs. malignancy).

Gender, age higher than 65 years, and length of hospitalization were not independently, significantly associated with the development of postoperative SSI. Operative approach, urgency of operation, smoking, diabetes mellitus, BMI, and TNM levels did not prove to have statistically significant association in the initial, cumulative model, and therefore were not included in the final model. The final ROC curve and AUC can be seen in Supplementary Figure S1. The multivariable logistic regression model was able to predict the occurrence of postoperative surgical site infection with satisfying accuracy following the bootstrapping process. The pseudo-*R*^2^ (McFadden's) confirmed a relatively good fit of our model to the data, and the final model was the one with the optimal AIC value (Table 3), further confirming that this is the optimal parameter selection (from the available) to predict SSI utilizing BChE levels. The overall accuracy of the final model is 0.952 with a sensitivity of 0.978 and a specificity of 0.852. The model's AUC (area under the curve) was calculated as 0.981 (Table 3), indicating that it has good predictive capability for the outcome of interest.

### 3.5 Butyrylcholinesterase levels at different time points between patients with any form of sepsis including secondary to anastomotic leak

When examining the relationship between BChE and the development of any type of infectious/septic complication, the differences do not appear to be statistically significant in favor of any patient group (see Table 4). Therefore, based on the univariate analysis, BChE does not seem to correlate with septic complications when they are grouped together.

**Table 4:** Univariate analysis of serum BuChE levels in septic complications.

BuChE value	Group	Mean	SD	*p*-value
BuChE (day of operation)	Septic complications	5.23	1.39	0.808
	Uncomplicated	5.16	1.28	
BuChE (first postoperative day)	Septic complications	4.73	1.27	0.649
	Uncomplicated	4.61	1.25	
BuChE (third postoperative day)	Septic complications	4.65	1.23	0.672
	Uncomplicated	4.54	1.20	
BuChE (fifth postoperative day)	Septic complications	4.82	1.21	0.725
	Uncomplicated	4.73	1.22	

The same pattern is observed when one examines the mean BChE levels in the serum of patients with postoperative anastomotic leakage (see Table 5). We observe that the BChE levels in patients with leakage did not significantly differ from those of uncomplicated patients.

**Table 5:** Univariate analysis of serum BuChE levels in anastomotic leak patients.

BuChE value	Group	Mean	SD	*p*-value
BuChE (day of operation)	Leak	5.36	1.39	0.584
	Uncomplicated	5.16	1.28	
BuChE (first postoperative day)	Leak	4.94	1.31	0.903
	Uncomplicated	4.61	1.25	
BuChE (third postoperative day)	Leak	4.81	1.16	0.818
	Uncomplicated	4.54	1.20	
BuChE (fifth postoperative day)	Leak	4.88	1.20	0.433
	Uncomplicated	4.73	1.22	

The association between BChE levels and the development of any form of sepsis on the first, third,^,^ and fifth day after surgery were also evaluated. BChE levels in patients with sepsis did not show statistically significant differences compared to uncomplicated patients during any time point postsurgery between patients with any form of sepsis and the cohort that did not (4.94 vs. 4.6, *p* = 0.903 4.81 vs. 4.54, *p* = 0.818 and 4.88 vs. 4.73, *p* = 0.433, respectively; tested with Mann–Whitney *U*-test and Welch's *t*-test).

### 3.6 Butyrylcholinesterase levels at different time points between patients with complications and uncomplicated patients

On the day of surgery and the first postoperative day, patients with any complication exhibited higher mean BChE levels although this difference was not statistically significant (5.31 KU/L vs. 5.16 KU/L, *p*-value = 0.438; and 4.68 KU/L vs. 4.61 KU/L; *p*-value = 0.640, respectively). During the third and fifth day postsurgery, a statistically significant difference was found in the mean serum BChE levels between patients with any complication and those without. Patients had significantly lower mean BChE levels (4.22 KU/L vs. 4.54 KU/L *p*-value = 0.015, and 4.45 KU/L vs. 4.73 KU/L, *p*-value = 0.029, respectively).

## 4 Discussion

To the best of our knowledge, this study is the first to try to evaluate the association between BChE levels and SSI after colorectal surgery in a prospective manner. The present study reveals that when adjusting for established risk factors for the development of SSI, low and decreasing BChE levels on the first and third day after colorectal surgery are correlated with an increased risk for SSI.

Various biomarkers for inflammation have been proposed, yet none have proven sufficient for early, specific, and accurate diagnosis of systemic inflammation (22). In this domain, BChE has recently been proposed as a diagnostic marker for low-grade systemic inflammation (23–25). Rapid changes in cholinesterase activity usually occur in patients following trauma, infections, burns, and critical illness (26–29). Both enzymes may act as systemic inflammation indicators and have potential prognostic value for mortality in critically ill patients. Zivkovic et al. (30) demonstrated that reduced BChE activity indicates severe systemic inflammation in critically ill patients. In this domain, a recent study indicated a prolonged reduction in serum cholinesterase activity predicts patient outcomes after sepsis (31).

The added benefit of the evaluation of BChE serves as a cost-effective, readily available laboratory indicator routinely measured. BChE activity is considered a surrogate parameter for the general clinical conditions of patients (32, 33). A study with 4,077 patients confirmed the role of BChE as an indicator of nutritional status and hepatic function (34, 35). To add to the increasing wealth of evidence, other studies have also supported low preoperative plasma cholinesterase activity as a risk factor for postoperative complications in the elderly population (31). However, it has been demonstrated that lower BChE levels correlate with complications and inflammatory conditions (36, 37). In a study of 453 patients, BChE was negatively correlated with complications, sepsis, and changes in nutritional status (38). BChE was directly correlated with leukocyte count and inversely correlated with bilirubin and sepsis (*p-*value < 0.01). Postoperatively, BChE decreased to 60% of preoperative values, remaining directly connected and decreasing further with sepsis. A study on patients with septic shock revealed a significant reduction in BChE levels compared to healthy controls (*p-*value < 0.01) (39–41). Survival rates were higher in patients with higher BChE levels. These results were also translated in the present study featuring colorectal surgery patients since low BChE levels on the first and third day after surgery were independent risk factors and correlated with increased odds for SSI.

Postoperative complications are not the only domain in which cholinesterase activity and levels should be studied in the surgical patient. A recent study looking into the BioCog patients concluded that a decrease in BchE activity was noticed more prominently in patients with postoperative delirium and complications, as opposed to uncomplicated patients (42). This is in line with the findings of our study that a decrease in BChE levels is strongly associated with postoperative septic complications. One hypothesis would be that the team's observations regarding postoperative delirium could be partially attributed to an underlying increase in the systemic inflammatory response, as heralded by a septic complication. Cholinesterase levels were lower in adults admitted to the ITU who exhibited signs of brain dysfunction and delirium, as seen in several studies (43–45). Lower BChE plasma levels were also successfully associated with worse cancer-specific prognosis, in a cohort of pancreatic cancer patients (46). In this 2020 study, the authors managed to associate the BChE plasma levels independently with pancreatic carcinoma survival rates in a single—institution study. Additional studies have also indicated that BChE is negatively associated with survival in various other cancers, such as renal cell, urothelial, and cervical carcinoma.

Another prime example of the utilization of BChE in alimentary tract carcinomas comes from the study of Gensthaler et al. (47), looking at baseline BChE levels in patients with resectable adenocarcinoma of the gastroesophageal junction. In this study, the authors also utilized multivariable regression modeling, in which BChE levels were negatively associated with overall survival and disease-free survival in patients. Although not being used in conjunction with postoperative outcomes, the authors in this study also commented on the possible association of lower BChE serum levels and an increase in systemic inflammation, as is our hypothesis. Furthermore, in both aforementioned studies, it would be interesting to see the possible differentiation of patients with postoperative complications, as they are both expected to have lower survival rates and possibly lower BChE plasma levels as well, therefore driving the initial observation of a strong correlation between BChE levels and overall survival in general. A smaller 2021 cohort study investigated the correlation between BChE and postoperative complications, only this time in patients after transcatheter aortic valve replacement (TAVI) surgery (38). Utilizing point-of-care measurements, the researchers were able to prove and present a strong association between BChE levels and complications after TAVI operations. This is indicative that the decrease in plasma BChE levels can be successfully used as an acute phase reactant biomarker, in response to a variety of postoperative complications, including in extra-abdominal procedures. In addition to septic postoperative complications, and postoperative delirium, the authors identified a strong correlation with the development of heart rhythm disturbances. Therefore, the results of the investigators are in line with our observations that an early decrease in BChE levels can be a herald of systemic inflammation, strongly correlated with postoperative complications. These observations can be extended beyond the qualitative approach and into quantitative observations. A recent study indicated that a higher decrease in plasma BChE levels independently correlated with worse patient outcomes, in burn patients (48), with the authors hypothesizing that the decrease in plasma BChE levels is directly proportional to the magnitude of the systemic inflammatory response.

The present study is not without limitations. Firstly, due to its observational nature, it carries the inherent patient allocation bias, and not every confounder has been accounted for, as would be expected if it was randomized. Similarly, there was a lack of a control group to also aid the adjustment for extra confounders. Future study designs by our research group intend to conduct prospective studies that will factor in more postoperative outcomes in colorectal patients, such as stroke and postoperative delirium since cholinesterase levels seem to be closely related to the neurological function of postoperative patients. In addition, the present study's unique research question makes it difficult to assume the correct sample size for the necessary sample size and power that it would need to reject the null research hypotheses and in other time points, though a positive correlation was indeed found and represents real-world data. To evaluate the predictive capabilities of the final model built as a result of this study, we intend to run a second, validation study with an independent cohort of postoperative colorectal patients. It is with this validation study that we intend to establish a cutoff value that could potentially be used in future clinical practice, as an evaluator of early postoperative complications in colorectal surgery. Future research efforts should explore a stronger correlation of BChE with specific inflammatory conditions in postoperative patients.

In conclusion, in this prospective observational study, low BChE levels in the first and third day postsurgery were associated with an increased risk for the development of SSIs but not sepsis. Further prospective studies are still needed and should be conducted to confirm these findings.

## Data Availability

The raw data supporting the conclusions of this article will be made available by the authors, without undue reservation.
