# Supplementary material for: Butyrylcholinesterase levels correlate with surgical site infection risk and severity after colorectal surgery: a prospective single-center study
Source: Front Surg. 2024 Aug 20;11:1379410. doi: 10.3389/fsurg.2024.1379410 (PMC11368738; doi:10.3389/fsurg.2024.1379410)
Supplement: Supplementary file 1 [file Datasheet1.docx]

**Supplementary Table 1**: Preoperative diagnoses of patients

|  | | | | | | | |
| --- | --- | --- | --- | --- | --- | --- | --- |
|  |  |  |  |  |  |  |  |
| **Diagnosis** | | **Counts** | | **% of Total** | | **Cumulative %** | |
| Anal Ca |  | 5 |  | 1.2 % |  | 1.2 % |  |
| Anastomotic Leak |  | 1 |  | 0.2 % |  | 1.5 % |  |
| Anastomotic Stenosis |  | 1 |  | 0.2 % |  | 1.7 % |  |
| Appendiceal Carcinoma |  | 2 |  | 0.5 % |  | 2.2 % |  |
| Bowel Ischemia |  | 30 |  | 7.5 % |  | 9.7 % |  |
| Bowel Obstruction |  | 4 |  | 1.0 % |  | 10.7 % |  |
| Bowel Perforation |  | 25 |  | 6.2 % |  | 16.9 % |  |
| Cancer Recurrence |  | 1 |  | 0.2 % |  | 17.2 % |  |
| Cecal Ca |  | 50 |  | 12.4 % |  | 29.6 % |  |
| Crohn's |  | 7 |  | 1.7 % |  | 31.3 % |  |
| Diverticulitis |  | 20 |  | 5.0 % |  | 36.3 % |  |
| Entaglement |  | 1 |  | 0.2 % |  | 36.6 % |  |
| Enterocolic Fistula |  | 1 |  | 0.2 % |  | 36.8 % |  |
| Fistula |  | 1 |  | 0.2 % |  | 37.1 % |  |
| Hernia |  | 1 |  | 0.2 % |  | 37.3 % |  |
| Left Colon Ca |  | 15 |  | 3.7 % |  | 41.0 % |  |
| Orthosigmoid Ca |  | 20 |  | 5.0 % |  | 46.0 % |  |
| Ostomy Reversal |  | 3 |  | 0.7 % |  | 46.8 % |  |
| Polyposis |  | 7 |  | 1.7 % |  | 48.5 % |  |
| Rectal Ca |  | 58 |  | 14.4 % |  | 62.9 % |  |
| Rectal Prolapse |  | 1 |  | 0.2 % |  | 63.2 % |  |
| Recurrence |  | 7 |  | 1.7 % |  | 64.9 % |  |
| Right Colon Ca |  | 56 |  | 13.9 % |  | 78.9 % |  |
| Sigmoid Ca |  | 65 |  | 16.2 % |  | 95.0 % |  |
| Sigmoid Stenosis |  | 5 |  | 1.2 % |  | 96.3 % |  |
| Toxic Megacolon |  | 1 |  | 0.2 % |  | 96.5 % |  |
| Transverse Colon Ca |  | 9 |  | 2.2 % |  | 98.8 % |  |
| Ulcerative Colitis |  | 5 |  | 1.2 % |  | 100.0 % |  |
|  | | | | | | | |


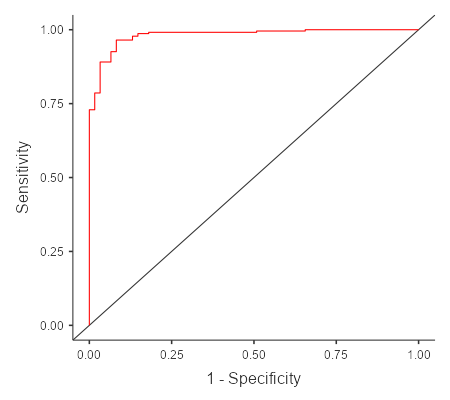


Supplemental Figure 1: ROC curve of the BChE – based predictive model
